# Inappropriate infant feeding practices of HIV-positive mothers attending PMTCT services in Oromia regional state, Ethiopia: a cross-sectional study

**DOI:** 10.1186/s13006-018-0181-x

**Published:** 2018-08-17

**Authors:** Daba Ejara, Demelash Mulualem, Samson Gebremedhin

**Affiliations:** 1 Meda Welabu University, Shashamene Campus, Shashamene, Ethiopia; 2 School of Nutrition, Food Science and Technology, Hawassa University, Hawassa, Ethiopia; 3 School of Public Health, Hawassa University, Hawassa, Ethiopia

**Keywords:** HIV exposed infants, HIV, Infant feeding practice, Exclusive breastfeeding

## Abstract

**Background:**

Inappropriate infant feeding affects the probability of mother-to-child transmission of HIV and HIV-free survival of infants. However, in Ethiopia limited evidence exists regarding the infant feeding practice of mothers who are HIV-positive. The aim of this study was to determine the prevalence and predictors of inappropriate infant feeding among HIV-positive mothers attending the prevention of mother-to-child transmission (PMTCT) service in Adama and Bushoftu towns, Oromia, central Ethiopia**.**

**Methods:**

A cross-sectional study was conducted in ten PMTCT service providing health facilities in the towns; 283 mother-infant pairs were enrolled. Appropriate infant feeding practice was defined as exclusive breastfeeding in the first six months of age. Logistic regression was employed to analyze the data and the outputs are presented using adjusted odds ratio (AOR) with 95% confidence intervals (CI).

**Results:**

One hundred thirty of the infants were aged below six months, 103 between 6 and 11 months and 50 were older than 12 months. The prevalence of inappropriate infant feeding was 14.5% (95% CI 10.6, 18.7). About 6.3% and 8.3% practiced exclusive replacement feeding and mixed feeding respectively, in the first six months. Only 1.8% ever expressed their breast milk to feed their baby and none practiced wet nursing. Among 38 mothers who already discontinued breastfeeding 52.6% did so before 12 months of age. Mothers who were HIV-positive and had received antenatal (AOR = 0.05: 0.01, 0.30) and postnatal visits (AOR = 0.18: 0.04, 0.81); received infant feeding counseling (AOR = 0.18: 0.06, 0.55); and disclosed their HIV status to their partners (AOR = 0.28: 0.12, 0.63), showed a reduction of practicing inappropriate infant feeding. Mothers having breast problems (AOR = 4.89: 1.54, 15.60) and infants with mouth ulcers (AOR = 6.41: 2.07, 19.85) were more likely to practice inappropriate feeding.

**Conclusion:**

Prompt management of breast complaints in mothers and mouth ulcer in infants; and provision of nutrition counseling to HIV-positive mothers, especially during antenatal and postnatal care, may help to improve the infant feeding practices for HIV exposed infants.

## Background

HIV/AIDS is a global pandemic affecting 36.7 million people including 2.1 million children under the age of 15 years [1, 2]. In 2016 there were about a million deaths from AIDS [3].

The Sub-Saharan Africa is the hardest hit by the pandemic with nearly two-thirds of all HIV cases and AIDS-related deaths [3]. The principal mode of HIV transmission is through unprotected sexual contact. Other causes include mother-to-child transmission, use of contaminated blood products for transfusion, and sharing of contaminated sharp objects [2, 3].

Mother-to-child transmission of HIV is the spread of HIV from an HIV-infected woman to her child during pregnancy, childbirth, or through breastfeeding [2] and studies have shown that in the absence of any intervention; between 20 and 45% of HIV-positive women transmit HIV to their babies during pregnancy, delivery, or through breastfeeding [2, 4]. The HIV transmission rate is estimated to be about 5–10% during pregnancy, 10–15% during labour and delivery, and 5–20% during the postnatal period through breastfeeding [2, 4].

While breastfeeding provides multifold health benefits to the infant, if the mother is not receiving antiretroviral therapy (ART), HIV can be transmitted during breastfeeding and the risk of transmission is proportional to duration of breastfeeding. The reduction of this transmission is one of the most pressing public health dilemmas confronting researchers, healthcare professionals, health policy-makers and HIV-infected women in many areas of the world [2]. Infant feeding practices recommended to women with HIV should support the greatest likelihood of HIV-free survival of their children. To achieve this, prioritization of prevention of HIV transmission needs to be balanced with meeting the nutritional requirements and protection of infants against non-HIV morbidity and mortality [5].

Based on the latest scientific evidence, the World Health Organization (WHO) revised its guideline on HIV and infant feeding in 2010. Further updates have also been made in 2013 and again in 2016 [6–9]. The 2010 guideline recommended, in settings where breastfeeding is judged to be the safest infant feeding option, mothers with HIV should breastfeed their baby exclusively for the first six months and continue to breastfeed for at least 12 months while introducing complementary foods [7]. In addition, all pregnant and lactating HIV-positive mothers should receive a lifelong ART regardless of their CD4 count, WHO clinical staging or gestational age. HIV exposed infants (HEIs) should also receive antiretroviral prophylaxis for 6–12 weeks [7, 8]. The recent 2016 WHO update recommends mothers living with HIV should breastfeed for at least 12 months and may continue breastfeeding for up to 24 months or beyond given they are receiving ART [9]. In the first six months of age exclusive replacement feeding should not be used unless safe and sufficient formula feeds are available [7–9].

Provision of ART to all pregnant and breastfeeding women living with HIV regardless of their immunological status, has changed the landscape of infant feeding recommendations in the context of HIV. ART protects infants against HIV transmission through breastfeeding even if the infant is breastfed beyond 24 months. Hence HEIs can maximize upon the benefits of exclusive breastfeeding in the first six months and prolonged breastfeeding later on. Further, ART reduces the risk of postnatal HIV transmission even in the context of mixed feeding [9].

According to the WHO, exclusive breastfeeding of HEIs for the first six months is vital for protecting them against common childhood illnesses including diarrhea and pneumonia [7–9]. Further, it promotes growth and development of the infant. Exclusive breastfeeding is much more effective than mixed feeding at protecting against common childhood illnesses, and mixed feeding many increase the risk of HIV transmission by 3–4 folds than exclusive breastfeeding [6]. Avoiding all breast milk and practicing exclusive replacement feeding is an option for preventing the risk of HIV transmission. However, this is less feasible in developing countries where access to safe and sufficient replacement feeding is limited. Further, replacement feeding deters the baby from maximizing upon the benefits of exclusive breastfeeding and may increase the risk of death from non-HIV causes [9].

Ethiopia is one of the high HIV/AIDS burden countries. According to the recent estimate, the HIV prevalence among adults age 15–49 years is 1.1% and the figure is as high as 5% in major urban areas [10]. As of 2017, an estimated 659,735 people were living with HIV/AIDS, of which 3.4% are children under the age of five years [10]. About 95% of the children in Ethiopia acquire the virus through mother-to-child transmission [11]. Adama and Bishoftu are among the towns with the highest rates of HIV prevalence in Ethiopia. The prevalence of HIV among pregnant women in the towns is 2.5 and 1.5%, respectively [12].

In Ethiopia limited information exists regarding the infant feeding practice of mothers who are HIV-positive. However, the few available studies indicate the practice is largely suboptimal [13, 14]. Accordingly, the purpose of the current study is to assess prevalence and predictors of inappropriate infant feeding practices among HIV-positive mothers attending the PMTCT service and receiving ART in Adama and Bushoftu towns. In this study, inappropriate infant feeding practice was considered as provision of non-exclusive breastfeeding in the first six months of age. The study considered both mixed feeding and exclusive replacement feeding as inappropriate practices due to the reasons discussed above.

## Methods

### Study setting

The study was conducted from January to March 2016, in Adama and Bishoftu towns, Oromia regional state, Ethiopia. The estimated population sizes of the towns in 2014 were 311,483 and 133,641, respectively. According to 2013/14 annual report of the regional health bureau, the HIV prevalence in Adama was 4.7% and 9872 HIV-positive clients were receiving ART. Similarly, Bishoftu had 1.2% prevalence of HIV and 3524 clients were on ART [12]. In the towns more than half (56%) of the sero-positive population are women. During the survey, 611 HIV-positive mothers in Adama and 243 in Bishoftu were attending PMTCT service, and there were 854 HEI [12]. Adama town has one government hospital and five health centers while Bishoftu town has one hospital and three health centers providing PMTCT services.

In both cities, in line with the national standard, PMTCT service is integrated with maternal, newborn and child health services. Antenatal care (ANC), postnatal care (PNC), family planning, ART, early infant diagnosis and ongoing counseling for infant feeding are all provided in one unit in integrated manner. During antenatal care, pregnant women received the HIV counseling and testing service and all HIV positive pregnant women are initiated on lifelong ART regardless of their immunologic status. During ANC and PNC visits they are provided ongoing continuous counseling about PMTCT including infant feeding options. Further, the integrated newborn and child care, provides an early infant diagnosis service for HIV exposed infants [13, 15].

### Study design

An institutional based cross-sectional quantitative study with both descriptive and analytic elements was conducted in PMTCT service providing health institutions in Adama and Bishoftu towns.

### Study participants

All mothers on ART who have HEI less than the age of 18 months and attending PMTCT services in public health institutions found in the two towns during the study period were considered eligible for the study.

### Sample size determination

Sample size adequate for determining the prevalence of non-exclusive breastfeeding among infants was computed using single population proportion formula with finite source population correction [16]. In the calculation 95% confidence level, 5% margin of error and 10% compensation for possible non-response were assumed. To maximize the sample size, the expected proportion was taken as 50% and ultimately sample size of 292 was determined. The adequacy of the sample size for identifying selected predictors of infant feeding practices was evaluated using post-hoc power analysis.

### Sampling technique

Stratified sampling technique was employed to select the study participant from all public health facilities found in the two towns. First sampling frame of 854 mothers with HEIs who were attending PMTCT service was obtained from registration books of each health facility. Proportion-to-size allocation was carried out to distribute the total sample size (*n* = 292) to all 10 public health institution providing PMTCT service in the towns. Ultimately, simple random sample was used to select the study subjects.

### Data collection

Data were collected by thirteen trained health professionals using pretested structured questionnaire. The questionnaire was prepared in English and translated into two local languages (Afan Oromo and Amharic). Medical records were reviewed and relevant information, including CD4 count and WHO clinical staging, were extracted.

Exclusive breastfeeding practice of the study participants was assessed by asking the mothers whether they have provided anything different from breast milk to the baby in the first six months of birth. For infants younger than six months, exclusive breastfeeding practice until the time of interview was appraised. In order to facilitate memory, the mothers were further asked whether the infant had received plain water, water-based fluids, non-human milk or fruit juices in the first six months of age or not.

The height and weight of the mothers and their infants were measured following standard procedures. Weight was measured with light clothing and without shoes using digital SECA scales. Infants’ height was measured in lying position without shoes using portable measuring boards.

### Study variables

The dependent variable of the study was feeding practice of HEIs categorized into two levels: appropriate or inappropriate. HIV-positive mothers who provided non-exclusive breastfeeding to their infants up to six months of age, including mixed feeding and exclusive replacement feeding, were considered as practicing inappropriate feeding [4, 17]. Replacement feeding was defined as feeding an infant who is not receiving any breast milk with a diet until it can be fully fed on family foods [9]. Mixed feeding was defined as an infant younger than six months of age who is given other liquids and/or foods together with breast milk [9].

The independent variables considered in the study include: sociodemographic and economic characteristics of the mother; maternal knowledge and attitude towards PMTCT and infant feeding recommendations; disclosure of HIV status; maternity services utilization during the index pregnancy; nutritional status and health condition of the mother including the presence of breast problems (engorgement, mastitis, breast abscess, nipple fissure/crack or breast thrush); progression of HIV and occurrence of mouth ulcer in the infant.

Progress of HIV was ranked into four stages (I-IV) based on the WHO clinical staging [8, 18]. Knowledge of HIV-positive mothers was assessed based on their knowledge about optimal duration of exclusive breastfeeding, total breastfeeding and timely introduction of complementary foods. Those who were aware that the HEIs should be exclusive breastfed for six months, commence complementary foods at the six months and continue breastfeeding for at least 12 months were considered knowledgeable. We considered mothers who know all three components knowledgeable.

The attitude of HIV-positive mothers on infant feeding was assessed based on five-points Likert scale and dichotomized into favorable and unfavorable categories using mean as a cutoff point. Attitude was assessed based on the following five statements: (*i*) HIV positive mother who opted for replacement feeding should breastfeed the infant in the presence of others; (*ii*) breastfeeding practice of HIV positive mothers contributes to the transmission of HIV to the baby; (*iii*) mother-to-child transmission of HIV/AIDS is unlikely to occur through breastfeeding; (*iv*) mixed feeding can minimize the risk of HIV/AIDS transmission; and (*v*) when the CD4 count is less, the risk of HIV transmission through breastfeeding increases.

In this study, we did not include ART status as an independent variable because, in accordance with the national guideline [13], all the study participants were on ART.

### Data management and analysis

Data were entered into Epi Info software and analyzed using SPSS. Frequency distributions, mean, standard deviation and tables were used to summarize the data. WAZ (weight-for-age), WLZ (weight-for-length) and LAZ (length-for-age) Z scores for HEIs were determined using the WHO anthro software. Predictors of infant feeding were identified using bivariable and multivariable logistic regression analyses. All the independent variables which showed *p* - value less than 0.25 in the bivariable models were considered as candidate variables for the multivariable model. In the multivariable model, absence of multicollinearity was assessed using variance inflation factor and the goodness-of-fit was measured by Hosmer and Lemeshow statistic.

## Results

### Sociodemographic and economic characteristics

A total of 283 HIV-positive mothers who had HEIs less 18 months of age participated in the study. Among those approached for the study, nine were not willing to take part accordingly the response rate of the study was 96.9%. The mean (± SD) age of the infants was 7.3 (± 4.3) months and about half (45.9%) were less than the age of six months. Slightly more girls (51.2%) than boys (48.8%) were represented in the study.

The mean (± SD) age of the mothers was 29.3 (± 4.0) years and 50.9% were younger than the age of 30 years. Three-fourths (76.3%) of the respondents were literate. Pertaining to occupation, more than two-thirds (68.9%) were daily laborers and 11.7% were housewives. About 66.8% had a monthly income of more than 1000 Ethiopian birr (approximately 36 USD). The major religious affiliations were Orthodox Christian (58.7%), Muslim (19.1%) and Protestant (18.7%); whereas the predominating ethnic groups were Oromo (48.4%) and Amhara (32.5%). The vast majority (90.8%) of the women were married (Table 1).

**Table 1:** Sociodemographic and economic characteristics of HIV-positive mothers following PMTCT service in public health facilities in Adama and Bishoftu towns, 2016

Sociodemographic characteristics (*n* = 283)	Frequency	Percent
Age of the mothers (Years)		
18–24	35	12.4
25–29	109	38.5
30–34	105	37.1
35+	34	12.0
Age of the infants (months)		
Less than 6	130	45.9
6–11	103	36.4
12–17	50	17.7
Sex of the infants		
Male	138	48.8
Female	145	51.2
Educational status of the mothers		
Unable to read or write /no formal education	67	23.7
Informal education and able to read or write	56	19.8
Grade 1–8	87	30.7
Grade 9–12	51	18.0
Tertiary education	22	7.8
Ethnic group		
Oromo	137	48.4
Amhara	92	32.5
Gurage	33	11.7
Tigray	13	4.6
Others	8	2.8
Religious affiliation		
Orthodox Christian	166	58.7
Muslim	54	19.1
Protestant	53	18.7
Catholic	10	3.5
Occupation of the mothers		
Daily laborers	195	68.9
Housewife	24	8.5
Private employee	23	8.1
Merchant	22	7.8
Government employee	10	3.5
House maid/servant	9	3.2
Marital status		
Married	257	90.8
Divorced/separated	18	6.4
Single	5	1.8
Widowed	3	1.1
Income (ETB)		
< 500	19	6.7
500–1000	75	26.5
> 1000	189	66.8

### Medical condition of the mothers and infants

Among the respondents 27.4% had a CD4 count less than 500 cells/mm^3^ and 50.9% were in WHO clinical stage I. About 8.1% of the mothers had encountered breast problems including breast engorgement (*n* = 10), sore nipples (*n* = 9) or burning and tingling (4) in the first six months after the birth of the index baby. Smaller proportions (5.7%) reported chronic diseases like tuberculosis, diabetes or hypertension. Among the total infants represented, 7.8% had developed mouth ulcer. For 233 (82.3%) of the infants HIV testing was done and 3.4% had positive test results. In remaining 50 HEIs, the mothers were yet to receive the test results of their baby (Table 2).

**Table 2:** Medical status of HIV-positive mothers and their infants receiving PMTCT service in public health facilities in Adama and Bishoftu towns, 2016

Medical conditions	Frequency	Percent
Maternal CD4 count (*n* = 281)		
< 500 cells/mm^3^	77	27.4
> 500 cells/mm^3^	204	72.6
Mother’s WHO clinical stage (*n* = 283)		
Stage I	144	50.9
Stage II	108	38.1
Stage III	18	6.4
Stage IV	13	4.6
Encountered breast problems (*n* = 283)		
Yes	23	8.1
No	260	91.9
Any chronic diseases (*n* = 283)		
Yes	16	5.7
No	267	94.3
Infant mouth ulcer (*n* = 283)		
Yes	22	7.8
No	261	92.2
Knows infant’s HIV status (*n* = 283)		
Yes	233	82.3
No	50	17.7
HIV status of the child (*n* = 233)		
Positive	8	3.4
Negative	225	96.6

### Nutritional status of the mother and infants

Nearly three-fourths of the study participants (74.2%) had a normal body mass index and 9.5% were wasted. The WAZ and WLZ for most infants, 82.7, 92.6% respectively, were within the normal (± 2 z-scores) ranges. Nevertheless, only 50.5% had normal LAZ score (Table 3).

**Table 3:** Nutritional status of the HIV-positive mothers and HEIs infants receiving PMTCT service in public health facilities of Adama and Bishoftu towns, January, 2016

Nutritional status (*n* = 283)	Frequency	Percent
Maternal body mass index (kg/m^2^)		
< 18.50 (wasted)	27	9.5
18.50–24.99 (normal)	210	74.2
> 25.00 (overweight or obese)	46	16.2
Weight-for-age Z score of the infant		
< −2	25	8.8
-2 to 2	234	82.7
> 2	24	8.5
Weight-for-length Z score of the infant		
< −2	11	3.9
-2 to 2	262	92.6
> 2	10	3.5
Length-for-age Z score of the infant		
< −2	97	34.3
-2 to 2	143	50.5
> 2	43	15.2

### Utilization of maternity services

The majority of the mothers (94.7%) had at least one antenatal care (ANC) visit during the index pregnancy and 67.9% had the recommended four or more visits. Most (90.8%) mothers gave birth at health institutions. Of the total, 84.1% delivered with spontaneous vaginal delivery and 8.8% had C-sections. The majority (94.3%) had at least one postnatal care (PNC) visit. During the pregnancy, 83.7% of them were counseled by health workers about infant feeding options. This includes counseling during antenatal care (90.5%), delivery (69.2%), postnatal care (63.3%) and ART visits (55.7%). Frequently discussed topics during the counseling were advantages of exclusive breastfeeding (84.8%) and risks associated with mixed feeding (74.6%).

### Knowledge about infant feeding options for HIV exposed infants

Knowledge of the mothers about infant feeding was assessed. The vast majority were aware of the optimal duration of breastfeeding (96.8%), optimal duration of total breastfeeding (96.1%) and the right time for the introduction of complementary foods (95.4%). Those who were aware that the infants should be exclusive breastfed for six months, commence complementary foods at six months and continue breastfeeding for at least 12 months were considered knowledgeable. In view of that, 93.0% of the mothers were rated knowledgeable.

### Infant feeding practices of HIV-positive mothers

Among 283 mothers who were HIV-positive, 94.3% ever breastfed their infants and 69.3% initiated breastfeeding within the recommended first hour of birth.

Almost nil (1.8%) ever expressed their breast milk to feed their baby and none practiced wet nursing. The practice of exclusive replacement was 6.7% and 8.3% practiced mixed feeding. Among mothers who practiced replacement feeding (*n* = 19), the major reasons for doing so were fear of HIV transmission via breast milk (*n* = 18) and perceived lack of breast milk (*n* = 5). On the other hand, justifications for mixed feeding include lack of knowledge about the harm of the practice (*n* = 18), illness of the baby (*n* = 8), advised by others (*n* = 6), maternal illness (*n* = 5) and fearing breast milk would be insufficient to the baby (*n* = 5).

Regarding the timing of complementary food introduction, about three-fourths (77.8%) of the mothers introduced solid foods when the child was six months old. However, 6.8% and 15.4% initiated it before and after six months, respectively.

At the time of the survey 38 (13.4%) of the mothers had already discontinued breastfeeding. Among them, 52.6% ceased breastfeeding early (before 12 months of age). The leading reasons for early cessation were: fear of HIV transmission (76.3%); advised by health workers to do so (7.9%), infant no longer wanted breastfeeding (5.3%), to encourage the baby to take more foods (5.3%) and mother was too sick to breastfeed (5.3%) (Table 4).

**Table 4:** Infant feeding practices of HIV-positive mothers receiving PMTCT service in public health facilities of Adama and Bishoftu towns, January, 2016

Infant feeding practices	0–5 months		6–11 months		12–17 months		0–17 months	
	Frequency	Percent	Frequency	Percent	Frequency	Percent	Frequency	Percent
Ever breastfed (*n* = 283)								
Yes	121	93.1	97	94.2	46	92	264	93.3
No	9	6.9	6	5.8	4	8	19	6.7
Time of initiation of breastfeeding (*n* = 283)								
Within first hour	91	70	66	64.1	39	78	196	69.3
After first hour	39	30	37	35.9	11	22	87	30.7
Ever gave expressed breast milk (*n* = 264)^a^								
Yes	2	1.6	2	2.1	1	2.3	5	1.8
No	124	98.4	93	97.9	42	97.7	259	98.2
Practiced exclusive replacement feeding (*n* = 283)								
Yes	8	6.2	7	6.8	4	8	19	6.7
No	122	93.8	96	93.2	46	92	264	93.3
Practiced mixed feeding (*n* = 264)^a^								
Yes	9	7.1	8	8.4	5	11.6	22	8.3
No	117	92.9	87	91.6	38	88.8	242	91.7
Breastfeeding the baby at the time of the survey (*n* = 264)^a^								
Yes	120	95.2	83	87.4	23	53.5	226	85.6
No	6	4.8	12	12.6	20	46.5	38	14.4
Age of child at cessation of breastfeeding (*n* = 38)^b^								
< 12 months	–	–	–	–	–	–	18	47.4
> 12 months	–	–	–	–	–	–	20	52.6

^a^Calculated among 264 mothers who ever breastfed their infants

^b^Calculated among 38 mothers who already discontinued breastfeeding at the time of the study

- Age disaggregated figures are not provided due to small denominators

### Inappropriate feeding practices of HIV-positive mothers

In this study mothers who provided non-exclusive breastfeeding for the first six months of life (mixed feeding or exclusive replacement feeding) were considered to have practiced inappropriate infant feeding. Among the total of 283 respondents the prevalence of such practice was 14.5% (95% CI 10.6, 18.7) and remaining 83.5% practiced exclusive breastfeeding. As indicated above the prevalence of exclusive replacement feeding and mixed feeding were 1.8% and 8.3%, respectively.

### Factors affecting infant feeding practice

The bivariable logistic regression analyses showed that infant feeding practice showed near to significant associations (*p* < 0.25) with maternal education, antenatal and postnatal visits, place of delivery, disclosure of HIV status to partner, receiving counseling on infant feeding options, attitude towards exclusive breastfeeding, maternal breast problem and infant mouth ulcer. In multivariable analysis seven variables emerged statistically significant (*p* < 0.05). These were; maternal attitude towards breastfeeding, maternal breast problem, infant mouth ulcer, disclosure of HIV status to partner, having antenatal and postnatal visits and receiving counseling on infant feeding options.

Mothers who were HIV-positive who had antenatal and postnatal care during the index pregnancy were 95% (AOR = 0.05, 95% CI 0.01, 0.30) and 82% (AOR = 0.18, 95% CI 0.04, 0.81) less likely to practice inappropriate infant feeding than their counterparts. Conversely, HEIs who developed mouth ulcer (AOR = 6.41, 95% CI 2.07, 19.85) and who were born to mothers with breast problems were more likely to practice inappropriate feeding (AOR = 4.89, 95% CI 1.54, 15.60). HIV-positive mothers who disclosed their HIV status were 72% less likely to practice inappropriate infant feeding (AOR = 0.28, 95% CI 0.12, 0.63). Mothers who had favorable attitude towards exclusive breastfeeding were 61% less likely to practice inappropriate feeding (AOR = 0.39, 95% CI 0.16, 0.90) than their counterparts (Table 5).

**Table 5:** Factors affecting infant feeding practices of HIV-positive mothers receiving PMTCT service in public health facilities of Adama and Bishoftu towns, 2016

Variables (*n* = 283)	Infant feeding practice		Odds ratio (95% confidence interval)	
	Inappropriate (*n* = 41)	Appropriate (*n* = 243)	Crude	Adjusted^a^
Attitude about exclusive breastfeeding				
Favorable	22	215	0.15 (0.07, 0.30)	0.39 (0.16, 0.97)^*^
Unfavorable	19	27	1^r^	1^r^
Mother had breast problem				
Yes	11	12	7.02 (2.85, 17.33)^*^	4.89 (1.54, 15.60)^*^
No	30	230	1^r^	1^r^
Infant had mouth ulcer				
Yes	14	8	15.17 (5.83, 39.44)^*^	6.41 (2.07, 19.85)^*^
No	27	234	1^r^	1^r^
Disclosure of HIV status to partner				
Yes	18	198	0.17 (0.09, 0.35)^*^	0.28 (0.12, 0.63)^*^
No	23	44	1^r^	1^r^
Mother’s educational status				
Unable to read or write	7	60	0.31 (0.92, 1.06)	0.09 (0.02, 0.45)
Informal education	13	43	0.81 (0.26, 2.48)	0.39 (0.11, 1.38)
Grade 1–8	8	79	0.27 (0.08, 0.89)	0.09 (0.02, 0.39)
Grade 9–12	7	44	0.42 (0.12, 1.45)	0.25(0.06, 0.98)
Tertiary education	6	16	1^r^	1^r^
At least one ANC visit during the pregnancy				
Yes	28	240	0.02 (0.01, 0.08)^*^	0.05(0.01, 0.30)^*^
No	13	2	1^r^	1^r^
Place of delivery				
Health institution	25	232	0.07 (0.03, 0.16)^*^	0.36 (0.09, 1.46)
Home	16	10	1^r^	1^r^
Counseled on infant feeding				
Yes	21	216	0.13 (0.06, 0.26)^*^	0.18 (0.06, 0.55)^*^
No	20	26	1^r^	1^r^
At least one PNC				
Yes	30	237	0.06 (0.02, 0.18)^*^	0.18 (0.04, 0.81)^*^
No	11	5	1^r^	1^r^

^*^Statistically significant association at *p* value of 0.05

1^r^ Set as the reference category

^a^Model adjusted for all the variables listed in the table

## Discussion

We surveyed the feeding practice of HEIs in two towns in central Ethiopia and found that the majority 85.5% of the mothers practiced appropriate feeding and the remaining 14.5% of mothers who were HIV-positive practiced inappropriate infant feeding, operationally defined as non-exclusive breastfeeding in the first six months of age. Having no antenatal and postnatal visits; failure to receive infant feeding counseling during the pregnancy; occurrences of infant mouth ulcer and maternal breast problems; were significant predictors of inappropriate feeding of HEIs.

The study considered exclusive replacement feeding in the first six months as inappropriate feeding practice based on the assumption that safe, sufficient and sustainable breast milk replacement feeding is not usually feasible in the developing world including Ethiopia. However, this assumption may not be strictly true and this may depend on the economic status of each household. Accordingly, the prevalence of inappropriate feeding might have been over estimated in our study.

The proportion of HIV-positive mothers practicing exclusive breastfeeding (85.5%) to their infants for the first six months was comparable with other local studies conducted in Mekele (90.3%) [13] and Gondar (83.8%) [19]. However, a study from a more urbanized setting in Addis Ababa reported a substantially lower figure (30.6%) [14]. Differences in the level of urbanization, socioeconomic status and recent changes in the national PMTCT guideline may have contributed for the observed variations. Studies from other African and developing countries reported lower exclusive breastfeeding rates: Uganda (24%) [20], Ghana (62%) [21], Zambia (35%) [22] and India (44%) [23]. This might be due to differences in exclusive breastfeeding practice in the general populations and variation in the economic accessibility breast milk replacements in the countries.

According to the WHO guidelines, breastfeeding with other foods or liquids in the first six month constitutes mixed feeding. The proportion of mixed feeding in this study was 8.3% which is comparable with the study done in Mekele (6.3%) [13] and Gondar (10.5%) [19], Ethiopia. The finding is lower than a study report in Addis Ababa, Ethiopia (15.3%) [1]. Likewise, studies from Ghana (40%) [21], Zambia (24.1%) [22] and India (29%) [23] reported higher figures.

We found that the rate of exclusive replacement feeding was 6.7%. A study in Mekele and Gondar found comparable figures, 3.4% and 5.7%, respectively [13, 19]. However, studies conducted in more urbanized settings in Addis Ababa, Ethiopia (46.8%) and South Africa (60%) reported substantially higher figs. [14, 24]. The low exclusive replacement feeding rate observed in our study could be due to the reason that most mothers had a low economic status and may not have been able to afford to buy breast milk substitutes.

In our study a few (1.4%) mothers who were HIV-positive ever used expressed and heat-treated breast milk to feed the baby. This might be due to the low cultural acceptability of this infant feeding option. Further, no mothers used wet-nursing, which is similar to that reported from Mekele, Addis Ababa and Gondar [13, 14, 19]. Likewise, the study done in Southern Ghana concluded that wet nursing and use of expressed heat-treated breast milk were neither culturally acceptable nor feasible infant feeding options [21].

Mixed feeding predisposes infants to increased risk of HIV transmission by damaging the integrity of the intestinal mucosa [7, 24]. Exclusive replacement feeding in settings where breast milk substitutes are not feasible may also lead to mixed feeding. In this study, the reasons for practicing mixed or exclusive replacement feedings were lack of knowledge on the associated risk, imposition by husband or influential others, perceived insufficiency of breast milk and maternal or infant illness. The same reasons were reported by two studies conducted in Ethiopia [14, 19]. In Addis Ababa, the reasons for mixed feeding were neighbor’s advice (40%), perceived insufficiency of breast milk (26%), mother’s illness (18%) and husband’s opinion (4%) [14]. In Gondar, 12.9%, and 9.6% of the mothers said that insufficient breast milk and husband opposition had led to mixed feeding [19]. The findings indicate, involving husbands in infant feeding and educating mothers that breast milk is sufficient to meet the nutritional requirements of the baby in the first six months may result in better feeding practices.

Mothers who were HIV-positive and who received antenatal and postnatal care were less likely to practice inappropriate infant feeding practice in contrast with their counterparts. Likewise, a study conducted in Tigray region, Ethiopia found that women who had ANC were 17 times more likely to practice recommended infant feeding practices [25]. A study conducted in Addis Ababa, Ethiopia and South Africa also came to the same conclusion [14, 24]. This may indicate that integration of nutritional counseling into antenatal and postnatal care can be an effective modality to promote optimal feeding of HEIs. The finding that mothers who received nutritional counseling are 82% less likely to practice non-exclusive breastfeeding in the first six months also supports this conclusion.

We found that mothers who were HIV-positive and having HEIs with mouth ulcer were six times more likely to practice inappropriate infant feeding practices as compared to their counterparts. Mouth thrush is painful and may interfere with effective suckling during breastfeeding. Infants may take short feed only or refuse to breastfeed. Hence mothers are likely to give other foods or fluids.

Breast problems like mastitis, abscess and cracked nipples are known to be associated increased breast milk HIV load and mother-to-child transmission of HIV [26, 27]. In our study about 8% of the mothers reported breast problems like engorgement and sore nipples during lactation and such occurrences are associated with mixed feeding. The finding may indicate that mothers who are HIV-positive need to be counseled about management of common problem during lactation and what to do when such problems arise.

In this study, mothers who disclosed their HIV-positive status had 72% reduced odds of practicing inappropriate infant feeding than their counterparts. This finding is consistent with a study conducted in Addis Ababa which indicated disclosure of HIV status to spouse made mothers 89% less likely to practice mixed feeding than those who didn’t [14]. According to a study conducted in Gondar, disclosure of HIV status with their spouse were found to have positive assocation with infant feeding practice [19]. If mothers didn’t disclose their status to their husbands, husbands may insist that mothers practice inappropriate infant feeding being not aware of risk of HIV transmission.

The reported level of exclusive breastfeeding can be under- or over-estimated as the practice was assessed retrospectively based on mothers’ recall. Restricting the study only to infants younger than six months would have reduced the recall error and might have allowed for more precise estimation of practice. Yet, due to the limited number of HEIs younger than six months available in the two towns we have to enroll older children too. Further, the exclusive breastfeeding rate might have been over estimated because nearly half (46%) of the study subjects were below the age of six months. Accordingly, such infants can still have the possibility of being exposed to non-exclusive breastfeeding before reaching the age of six months. In addition, social desirability bias could have also resulted in overestimation of the practice.

The findings of the study should be interpreted in consideration of the following limitations. The study was conducted among PMTCT clients of public health institutions, and the findings may not be generalizable to mothers utilizing service from private providers as differences in socioeconomic characteristics are likely. Recall errors might have also affected other responses including timely introduction complementary foods and cessation of breastfeeding. There is also a possibility that participants who received counseling on appropriate feeding practices may simply provide favorable responses and introduce reporting bias in the study. In the multivariable analysis, some of the table cells had fewer observations and this may have resulted in lower statistical power.

## Conclusion

The study found that smaller proportion (15%) of mothers who were HIV-positive practiced inappropriate infant feeding. Mothers who had antenatal and postnatal care, received counseling on infant feeding practices and disclosed their HIV status were less likely to practice inappropriate infant feeding practice. Whereas mothers whose infant developed a mouth ulcer and mothers with a breast problem were more likely to practice inappropriate infant feeding practices.

Prompt management of breast complaints in mothers and a mouth ulcer in infants, and provision of nutrition counseling to mothers who are HIV-positive especially during antenatal and postnatal visits may contribute to improving the infant feeding practices for HIV exposed infants.

## Abbreviations

ANC: Antenatal Care
AOR: Adjusted Odds Ratio
ART: Anti Retroviral Therapy
CI: Confidence Interval
EDHS: Ethiopian Demographic and Health Survey
ETB: Ethiopian Birr
FMOH: Federal Ministry of Health
HEIs: HIV Exposed Infants
LAZ: Length-for-age Z score
MTCT: Maternal-to-Child Transmission
ORHB: Oromia Regional Health Bureau
PMTCT: Prevention of Mother-to-Child Transmission
PNC: Postnatal Care
UNAIDS: United States Agency for International Development
WAZ: Weight-for-age Z score
WHO: World Health Organization
WLZ: Weight-for-length Z score
